# Effect of treatment on back pain and back extensor strength with a spinal orthosis in older women with osteoporosis: a randomized controlled trial

**DOI:** 10.1007/s11657-018-0555-0

**Published:** 2019-01-09

**Authors:** Christina Kaijser Alin, Elin Uzunel, Ann-Charlotte Grahn Kronhed, Hassan Alinaghizadeh, Helena Salminen

**Affiliations:** 1 Division of Family Medicine and Primary Care, department of Neurobiology, Care Sciences and Society, Karolinska Institutet, Solna, Sweden; 2 Rehab Väst, Local Health Care Services in the West of Östergötland, Östergötland, Sweden; 3 Division of Physiotherapy, Department of Medical and Health Sciences, Linköping University, Linköping, Sweden; 4 Academic Primary Health Care Centre Stockholm, Stockholm, Sweden

**Keywords:** Osteoporosis, Back pain, Spinal orthosis, Back extensor muscle strength, Vertebral fracture, Kyphosis

## Abstract

**Summary:**

The treatment effect of an activating spinal orthosis on back pain and back extensor strength was compared to a training group and to a control group. Between the groups, there was no significant difference in back pain, back extensor strength, or kyphosis index after the 6 months of treatment.

**Purpose:**

The aim of this study was to study the effect of treatment with an activating spinal orthosis on back pain, back extensor strength, and kyphotic index. Our hypothesis was that an activating spinal orthosis may be an alternative treatment to decrease back pain and increase back extensor strength.

**Methods:**

A total of 113 women aged ≥ 60 years with back pain and osteoporosis, with or without vertebral fractures, were randomized to three groups: a spinal orthosis group, an equipment training group, and a control group. All three groups were examined at baseline and followed up after 3 and 6 months. Statistical analyses were performed with a mixed model for repeated measures according to intention to treat (ITT) and per protocol (PP).

**Results:**

A total of 96 women completed the study. Between the groups, there was no significant difference in baseline characteristics. Comparison between groups showed no significant difference in back pain, back extensor strength, or kyphosis index at the follow-up after 6 months according to ITT and PP analyses. Analysis in each group showed that the back extensor strength had increased by 26.9% in the spinal orthosis group, by 22.1% in the exercise training group and by 9.9% in the control group.

**Conclusions:**

Six months’ treatment by an activating spinal orthosis showed no significant difference in back pain, back extensor strength, or kyphosis index between the three groups. In the spinal orthosis group, present back pain decreased slightly and back extensor strength increased by 26.9% which indicates that the spinal orthosis may become an alternative training method.

Clinicaltrials.com ID: NCT03263585

**Electronic supplementary material:**

The online version of this article (10.1007/s11657-018-0555-0) contains supplementary material, which is available to authorized users.

## Introduction

Osteoporosis is a common health problem among older women and often results in chronic back pain and reduced health-related quality of life, often as a consequence of vertebral fractures [1]. According to a survey conducted in 27 European Union (EU) states, there were 22 million individuals suffering from osteoporosis and over half a million new vertebral fractures were diagnosed during 2010. The estimated costs for incident and prior fragility fractures were 37 billion euros [2].

Vertebral fractures are the most common type of osteoporotic fractures, although it is estimated that only one third of them actually come to clinical diagnosis even if they are associated with back pain and limitations of daily life [3–5]. Vertebral fractures are also known to cause kyphosis of the thoracic and/or lumbar spine [6]. Kyphosis can also result in a reduction of the lung capacity [7]. Women suffering from osteoporosis have a lower quality of life than healthy women and it may be associated with increased thoracic kyphosis and reduced back muscle strength. Several studies are suggesting positive effects of exercise programs on pain, quality of life, and daily functioning in postmenopausal women and men with or without kyphosis and vertebral fractures and training of the back extensor muscles and posture training may also reduce the kyphosis and further be associated with the risk of future vertebral fractures [8–18].

In Sweden, the National Board of Health and Welfare published in 2012 national guidelines for the treatment of patients with osteoporosis. The compliance with these guidelines has been low. In Stockholm, at the time of this study, there was no usual care for patients who had suffered an osteoporotic fracture and very few women had access to rehabilitation programs. Women diagnosed with osteoporosis visiting any of the rehabilitation units in Stockholm City were offered training, either in group or individually, as well as a home exercise program and some of the rehabilitation units also offered an osteoporosis school.

An activating spinal orthosis has been developed for the treatment of patients with osteoporosis, vertebral fractures, and back pain. Promising results have shown an increase in back extensor muscle strength, improved posture, and positive effects on pain and activities of daily life, and also improved pulmonary function [19, 20].

Back pain is a common and limiting condition in older women that causes great suffering for the individual and high costs for the society. Therefore, it is important to further evaluate treatment alternatives. A spinal orthosis that strengthens the back extensor muscles via biofeedback could be a good alternative. The orthosis mentioned above [19, 20] has neither been evaluated among women with back pain and without recently occurred vertebral fractures nor compared to physiotherapy.

Our hypothesis was that wearing an activating spinal orthosis can be used as an alternative or additional treatment to equipment training to increase back extensor strength in older women suffering from osteoporosis and back pain.

The main aim of the present study was to compare the use of an activating spinal orthosis with physiotherapy equipment training, and with a control group on back pain, back extensor strength, and kyphotic index in older women with osteoporosis, independent of vertebral fracture status.

## Material and methods

### Study design

This study was a randomized controlled trial (RCT) with three groups: one group wearing an activating spinal orthosis, an equipment training group, and a control group.

### Participants

Inclusion criteria in the RCT were women aged ≥ 60 years, no upper age limit was set, and with diagnosed osteoporosis, back pain with or without vertebral fractures. The diagnosis of osteoporosis was self-reported but everyone was asked if bone mineral density was measured. Exclusion criteria were difficulties in following the research protocol and language problems, further diagnosed spinal stenosis because the experience of using the spinal orthosis may increase lumbar back pain in these patients.

The study was conducted from May 2012 to December 2014. Randomization was performed in four rounds. The first randomization started in May 2012, the second in February 2013, the third in October 2013, and the fourth and last one in June 2014. The intervention program lasted 6 months and all participants had follow-ups after 1 month, 3 months, and at the end of the intervention period.

The women who were recruited to the study came from three different populations. Women who participated in a follow-up study in the PRIMOS project (Primary Care and Osteoporosis) in 2012–2013 were invited. Women included from this population were born between 1920 and 1930 and lived in Bagarmossen, a suburb south of Stockholm. Thirteen women who met the inclusion criteria and were interested in participating in the RCT were randomized. Women who participated in an osteoporosis school at Rehab City Kungsholmen in Stockholm City between 2007 and 2010 were also invited and 15 women were randomized to the study population. The study was advertised in four local newspapers in Stockholm City and in a patient association newspaper, and 85 women were randomized to the study from these sources. All women lived in Stockholm County, and most women lived in Stockholm City. This minimized logistical problems for those women randomized to the training groups which took place at Rehab City Norrmalm and Rehab City Östermalm, two rehabilitation units in Stockholm City.

**Fig. 1:**
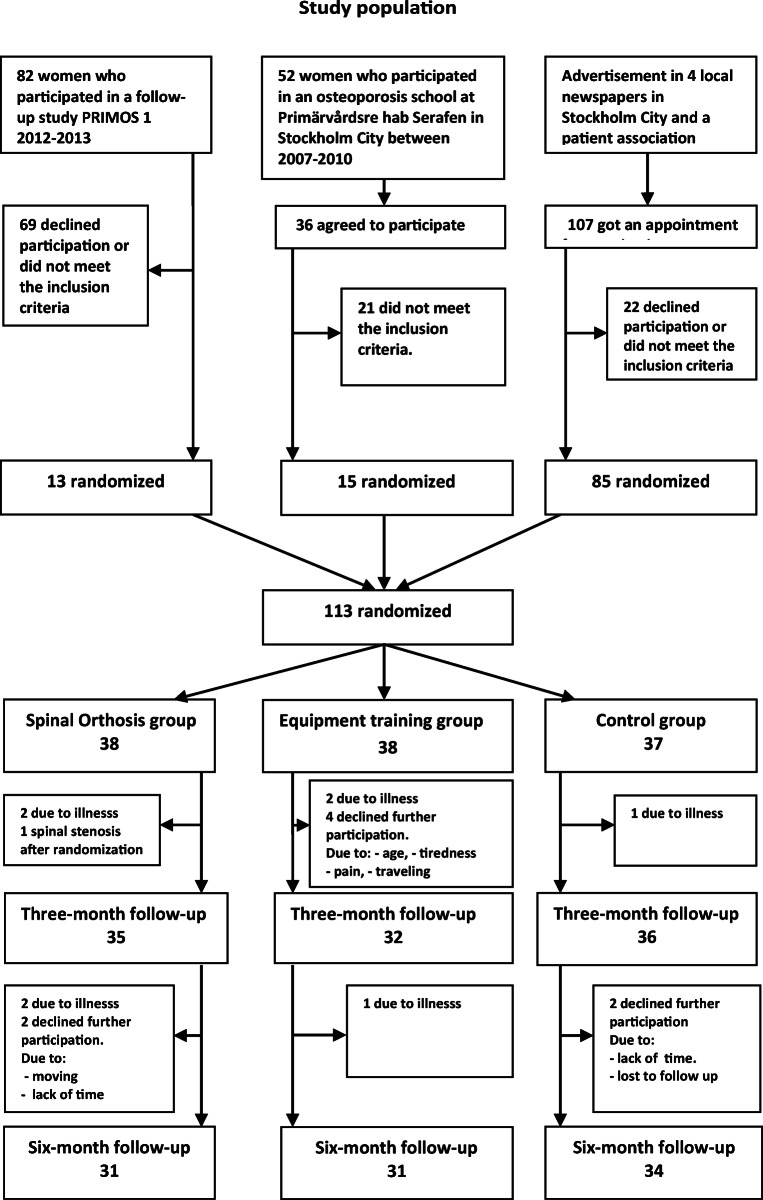
Flow chart of the participants in the RCT

More information on the study population can be found in Fig. 1.

### Intervention

Women who reported their interest to participate in our study received information about how the study was designed and that they would be randomized into one of the three groups.

#### The spinal orthosis group

Participants who were randomized to the spinal orthosis group to wear the activating spinal orthosis were to follow the recommendation given by the physician or the physiotherapist on how to use the orthosis and time wearing it. They were also given an appointment to an orthopedic technician for individual adjustment of the orthosis to the back. The participant could call the orthopedic technician at any time for an appointment during the study period of 6 months to adjust the orthosis, so it would fit well and feel comfortable. The women were told to wear the orthosis, both by the physiotherapist, the physician, and the orthopedic technician, for about 10 min a day for the first couple of days and then successively to increase time in the orthosis during the following 2 weeks. The aim was to have the participant wear the orthosis for a total of 2 h or more per day. The 2 h could be divided into shorter periods, such as half an hour in the morning, 1 h at noon, and another half an hour in the evening. They were also asked to keep a logbook and estimate the time wearing the orthosis per week and also adverse events, such as illness, could be noted in the logbook.

The orthosis that the participants in the study wore was the activating spinal orthosis Spinomed. The spinal orthosis is constructed with a steel rail in the back which passes from the C7 to the sacrum and is adapted to the spinal curvature in an upright standing position. The rail is plugged into a compartment on the orthosis. The orthosis is put on as a backpack with straps around the shoulders and fastened around the pelvis. When the person is wearing the orthosis and flexes the back, the rail that is adapted to the spinal curvature and the straps around the shoulders will act as a feed back to continuously activate the back extensor muscles. The lower part of the orthosis, which is fastened over the pelvis, provides support for the lumbar spine through the pressure that occurs when the orthosis is tightened across the lower part of the abdomen.

#### The equipment training group

Participants who were randomized to the equipment training group exercised 1 h once a week for 6 months at a gym at Rehab City Norrmalm or at Rehab City Östermalm, led by a physiotherapist. Equipment training involved training in gym with sequence training equipment according to an individually tailored exercise program. The training started with bicycling, crosstrainer, or treadmill as warming up. The exercise program included balance exercises on a carpet, balance plate, and exercises with a rubberband and bobathball. The equipment training program was focusing on training the back extensor muscles, training of the posture, balance, and muscle strength of the legs. Further, the participants were told to perform a home exercise program at least four times a week, also focusing on back extensor strength and balance. Participants were asked every week when they arrived at the equipment training group if they had performed the home exercise program. During the study period from May 2012 to December 2014, there were six different physiotherapists who led the equipment training group and they were familiar with how the training would be carried out.

#### The control group

The participants in the control group were assessed at baseline, 3 and 6 months, but no other intervention was given during the 6-month treatment period and they were asked to continue their ordinary life. When the intervention period was ended, they were offered training in an equipment training group and they also received a home exercise program.

### Measurements

The participants were examined by an experienced physiotherapist or a physician at Sabbatsberg Hospital in Stockholm. Back pain and back extensor strength were measured at baseline, after 3 months and after 6 months. The Visual Analogue Scale (VAS) where no pain was rated as 0 mm and worst possible pain as 100 mm and Borg CR-10 (0–10) were used to estimate back pain scores [21–24]. Participants were asked to score their present back pain and also to make an overall assessment of pain for the previous week.

Isometric back extensor strength was determined with the computerized device DigiMax (Mecha-Tronic, Germany) [19, 20, 25]. Participants were sitting in a fixed standardized position, fixed by a seatbelt around the hip and chest, with 90° in hip and knee and were asked to press the upper part of the body against a plate for 6 s. The results were presented as a mean pressure, in Newton, for 6 s and also as a maximum pressure at any time during the 6 s.

The spinal curvature was assessed at baseline and after 6 months. The spinal curvature was measured by the Flexicurve ruler (manufacturer Pedihealth AB, Finland). The Flexicurve ruler is flexible and is molded to the curve of the spine, with the participant standing in an upright position, and then traced on a paper. C7 and S1 vertebrae were located by manual palpation before applying the Flexicurve ruler [26–28].

To calculate the kyphotic index and angle, a reference line was drawn between C7 and S1 at the widest point on the both curves and the width of the curves and the length of the two halves were measured. The kyphotic index was calculated as the kyphosis width divided by the length times 100. The kyphosis measurements were split into the subgroups normal kyphosis and hyperkyphosis and the presence of hyperkyphosis was set at a clinically relevant cut-off point to ≥ 13 [29]. At baseline, a sagittal X-ray was taken of the thoracic and lumbar spine to investigate the presence of vertebral fractures. The X-ray was analyzed using the semi-quantitative Genant classification [30].

Baseline data were collected concerning age, marital status, housing, community care, home health care, diseases, falls in the past year, history of fractures as an adult, height at young adulthood age, and use of walking aid and medication concerning bone-specific drugs, calcium-vitamin D, only vitamin D, pain killers, corticosteroids, and inhalation of corticosteroids. Other questions concerned lifestyle: time spent outdoors, smoking, physical activity, and walking. Present body height was measured in centimeters by a stadiometer standing with the woman’s heels against the wall and the height loss since young adulthood was estimated. Weight was measured in kilograms. Hand grip strength of the dominant and the non-dominant hand was measured by the Jamar dynamometer in kilograms [31]. Spirometry (Welch Allyn, SpiroPerfect Spirometry, USA) was taken to assess forced vital capacity (FVC).

### Outcomes

Primary outcome was back pain assessed by VAS and Borg CR-10, measured at baseline, after 1 month, 3 months, and at the end of the intervention period, that is, at 6 months. Secondary outcomes were back extensor strength assessed by DigiMax and measured at baseline, after 3 months, and at the end of the intervention period, and spinal curvature assessed with Flexicurve ruler at baseline and at the end of the intervention period at 6 months.

### Sample size

Power calculation was based on the assumption that wearing an activating spinal orthosis could be used as a method comparable to an equipment training group to reduce back pain and to detect an evident difference compared to the control group. Power calculation was performed by G*Power [32] to detect differences in back pain change between the groups measured by VAS. With a sample size of 99 participants using medium effect size [33], 33 women in each group, and three measurement time, analyzed with ANCOVA, there was 88% power on alpha 5% level to detect a difference between groups. With an estimated dropout of 10%, we decided to include at least 36 women in each group.

### Randomization

Closed envelopes were used in randomization allocation, numbered from 1 to 113 and inside a patch showing the randomized group, i.e., spinal orthosis, exercise, or control group. The random allocation was performed by two experienced physiotherapists and two physicians who also enrolled and assigned participants to the interventions.

### Statistical analysis

Group results were reported as means and standard deviations for normally distributed continuous variables and as median with range for skewed distribution. One-way analysis of the variance (ANOVA) was used for comparisons of differences in variables between the three treatment groups at baseline for continuous variables and *χ*^2^ test for variables that were categories with variations in proportions. Paired *t* test was used to analyze change between baseline and 6-month follow-up in each group.

We analyzed the outcomes using a mixed model for repeated measures according to intention-to-treat procedure [34]. The dependent variables were back pain and back extensor strength measurements at baseline and after three and 6 months of treatment and spinal curvature measurements at baseline and after 6 months. The final results are presented in Table 3 by least square mean (LS mean) after adjustment for age, vertebral fractures, and FVC [35]. Both intention-to-treat analysis and a per-protocol analysis were performed.

**Fig. 2:**
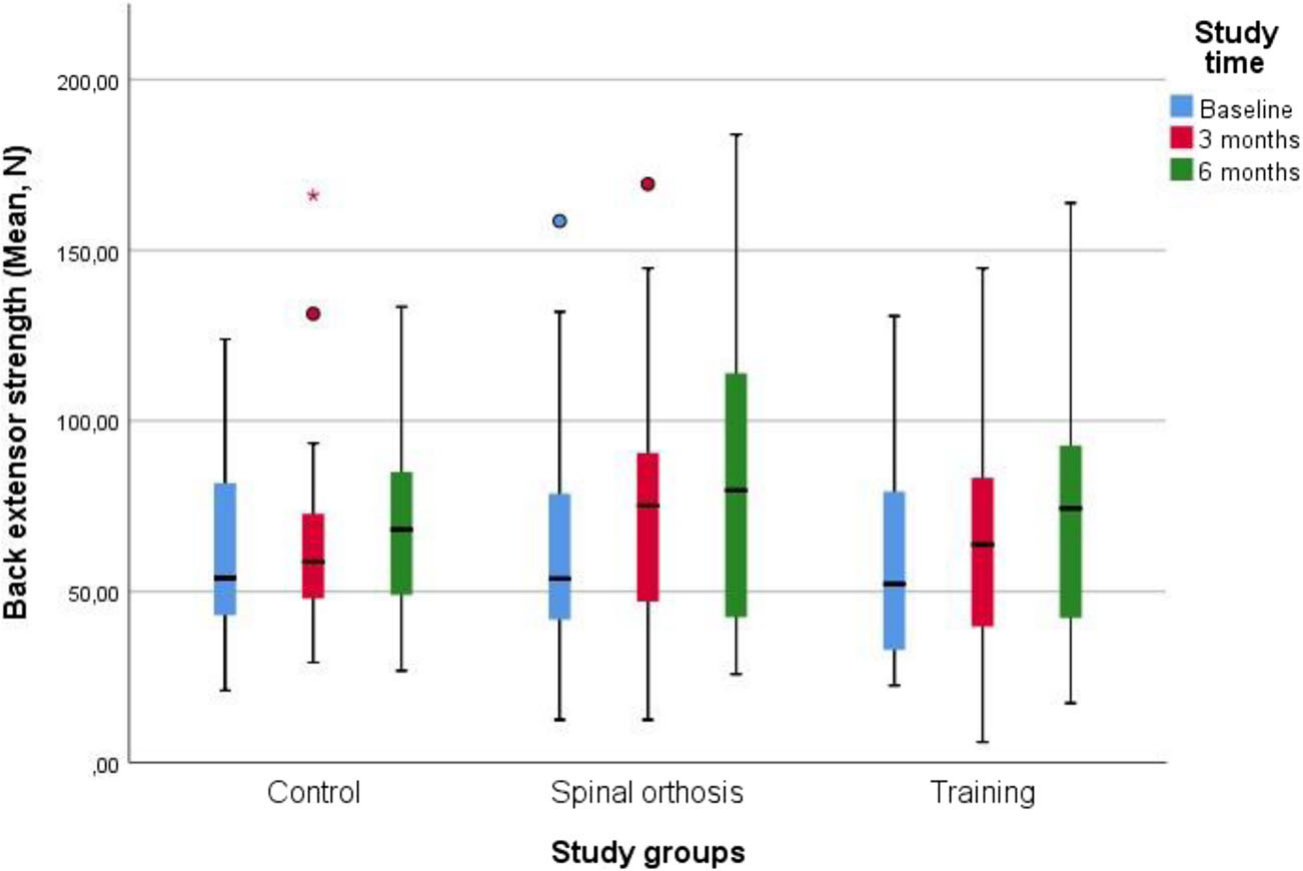
Box-plots of back extensor strength (mean, Newton) separated into study groups and study time

Significance levels below 5% were considered significant. The data were analyzed using the STATA, version 14, (StataCorp LP, Texas, USA) and SAS version 14. (SAS Institute, Cary, NC, USA). The analysis of Fig. 2 was done in the statistical program SPSS from raw data.

### Ethical consideration

Women who were interested in participating in the study received oral and comprehensive written information on what it meant to participate in a randomized controlled study and that participation was completely voluntary and could be terminated at any time. Written informed consent for participation was given. Ethical approval was obtained from the Ethical Review Board of Stockholm. Dnr 2011/142-31/3.

## Results

A total of 113 women were randomized and 96 women completed the RCT. Drop outs and reasons for drop outs are shown in Fig. 1. Median age was 76 years (IQR, 67–82 years). The age distribution between the groups was equivalent. Baseline characteristics of the study participants showed no significant difference between the groups for any variable besides present back pain measured by Borg CR-10 which was higher in the spinal orthosis group (*p* < 0.05). The number of women who self-reported a vertebral fracture was lower than could be identified on the X-rays. There were 13% more women who had vertebral fractures on X-rays that were not clinically known. Back extensor strength showed a large variance in the three groups. Back pain measured by VAS and Borg CR-10 both at current time and previous week showed a wide range. The participants estimated their back pain as weak (light) to very strong, with the median estimated score according to Borg CR-10 last week being 3 and VAS being 42 mm (moderate). The percentage of women who used pain killers when necessary was in the spinal orthosis group 21%, in the training group 10%, and in the control group 35%. No woman used pain killers regularly. The percentage of women with a kyphotic index ≥ 13 was about 50% in each group. Women treated with bone-specific drugs were in the spinal orthosis group 44.7%, in the training group 47.4% and in the control group 51.4%. A majority of the women were treated with calcium-vitamin D, 89.5% in the spinal orthosis group, 86.8% in the training group, and 89.2% in the control group. Baseline characteristics of the study participants are shown in Table 1.

**Table 1:** Baseline characteristics

	Spinal orthosis *n* = 38	Training *n* = 38	Control *n* = 37	
Variable	Mean (SD)	Mean (SD)	Mean (SD)	*p* value^c^
Present height (cm)	159.8 (7.6)	159.3 (7.6)	161.5 (6.9)	0.407
Height young (cm)	166.2 (5.7)	165.6 (6.3)	167.6 (5.7)	0.303
Weight (kg)	64.7 (13.4)	60.3 (8.5)	66.1 (11.6)	0.073
Back muscle extensor strength^a^ (N)	64.4 (32.8)	59.6 (30.8)	62.3 (25.2)	0.791
FVC^b^ (l)	2.7 (0.7)	2.6 (0.7)	2.7 (0.6)	0.727
Variable	Median (IQR)	Median (IQR)	Median (IQR)	*p* value^c^
Age	77.9 (68.0–83.4)	77.6 (67.3–84.1)	72.8 (67.5–78.2)	0.201
Visual Analogue Scale VAS back pain, recent (mm)	22 (10–40)	9 (1–30)	21 (10–45)	0.088
Visual Analogue Scale VAS back pain, last week (mm)	50 (28–69)	39 (20–52)	43 (20–61)	0.181
Borg CR-10 back pain, recent	3 (1–3)	2 (0.5–3)	2 (1–3)	0.049
Borg CR-10 back pain, last week	4 (2–7)	3 (2–4)	3 (3–5)	0.168
Variable	%	%	%	*p* value^d^
Kyphotic index ≥ 13	51.4	50.0	56.8	0.826
Bone-specific drugs	44.7	47.4	51.4	0.847
Calcium-vitamin D	89.5	86.8	89.2	0.925
Only vitamin D	2.6	5.3	2.7	0.784
Hip fracture self-reported	5.3	10.5	8.1	0.704
Wrist fracture self-reported	26.3	23.7	27.0	0.942
Vertebral fracture self-reported	36.8	31.6	24.3	0.508
Vertebral fracture X-ray	47.1	46.0	38.2	0.725

^a^Spinomed *n* = 35, training *n* = 38, control *n* = 36

^b^FVC forced vital capacity

^c^One-way ANOVA was used for comparisons of differences between the three treatment groups

^d^*χ*^2^ test for variables that were categories in proportions

During the intervention period, one new vertebral fracture occurred in one of the participants. More detailed information about adverse events is shown in the attached supplement (suppl Adverse events).

The attendance rate at the equipment training sessions was 70.3% for the 6-month period. According to the information provided at the follow-up, all women who completed the intervention period had been wearing the spinal orthosis at least 2 h per day.

In the activating spinal orthosis group, the back extensor strength increased by 26.9% and in the exercise training group by 22.1%. In the control group, back extensor strength increased by 9.9%, (Table 2).

**Table 2:** The change in percentage of back extensor strength in each group, analyzed by paired *t* test. Analyses per protocol

Baseline					6 months				
	Treatment group	*n*	mean ± SD	95% CI	*n*	mean ± SD	95% CI	Change %	*p* value
Muscle strength (N)	Spinal orthosis	25	64.4 ± 32.8	53.2–75.7	25	81.7 ± 41.3	65.4–98.0	26.9	0.053
	Training	30	59.6 ± 30.8	49.5–69.8	30	72.8 ± 37.3	58.9–86.8	22.1	0.013
	Control	31	62.3 ± 25.2	53.7–70.8	31	68.4 ± 27.0	58.7–78.1	9.9	0.153

Back pain measured by VAS and Borg CR-10 did not show any statistically significant changes between the three treatment groups. Isometric back extensor strength showed no significant difference between the three treatment groups and the kyphosis index did not show any significant changes between the three treatment groups after 6 months of intervention, see Fig. 2.

Comparison between the three treatment groups was analyzed according to intention to treat (ITT), unadjusted and adjusted for age, vertebral fractures, FVC, and examiner. Contrast tests were performed between the three groups as a post hoc analysis which showed no significant treatment effect. The result of the effect size (*f*^2^) based on Cohen’s guidelines [33] was 0.09 and could indicate that there was a small treatment effect but nevertheless the results from contrast tests showed no significant treatment effect between the groups (Table 3).

**Table 3:** Changes in back pain and back extensor strength between groups at baseline and at follow-ups after 3 and 6 months analyzed according to intention to treat and by mixed linear model, showing the group vs time interaction LS mean ± SE, *p* value, effect size and power of test, adjusted for age, vertebral fractures, FVC, and examiner

	Treatment group	*n*	Baseline mean ± SE^b^	3 months mean ± SE^b^	6 months mean ± SE^b^	*p* value	ES (*f*^2^)^a^	Power^c^
Back pain present VAS (mm)	Spinal orthosis	35	23.60 ± 4.07	20.00 ± 3.83	24.95 ± 3.93	< 0.01	0.08	0.92
	Training	37	14.24 ± 3.81	14.09 ± 3.71	18.69 ± 3.76			
	Control	35	24.87 ± 4.14	27–65 ± 3.72	21.55 ± 3.69			
Back pain present Borg CR-10	Spinal orthosis	35	2.16 ± 0.29	1.96 ± 0.30	1.78 ± 0.30	< 0.01	0.08	0.96
	Training	37	1.73 ± 0.27	1.36 ± 0.29	1.38 ± 0.28			
	Control	35	2.34 ± 0.28	2.36 ± 0.29	1.92 ± 0.28			
Muscle strength (N)	Spinal orthosis	35	65.89 ± 5.01	73.16 ± 5.15	82.48 ± 5.29	< 0.01	0.09	0.99
	Training	38	60.66 ± 4.82	67.32 ± 5.01	74.86 ± 5.04			
	Control	36	58.93 ± 4.99	61.70 ± 5.12	65.41 ± 5.09			

*p* value = interaction between time and treatment group

^a^Effect size (*f*^2^) is based on Cohen’s (1988) guidelines; *f*^2^ ≥ 0.02, *f*^2^ ≥ 0.15, and *f*^2^ ≥ 0.35 represent small, medium, and large effect size, respectively

^b^Estimated least square mean adjusted for age, vertebral fractures, and FVC and examiner

^c^Power of test showed that sample size was large enough to show a significant difference between the groups. Nevertheless, the contrast tests between the groups as a post hoc analysis showed no significant treatment effect

## Discussion

The aim of the study was to examine the effect of treatment on back pain, back extensor strength, and changes in spinal curvature by wearing the spinal orthosis compared to a physiotherapy equipment training group and a control group.

In this study, there was no significant difference between the spinal orthosis group, the equipment training group, and the control group regarding back pain, back extensor strength, and decreased kyphotic index. In the spinal orthosis group where the women have been wearing the activating spinal orthosis at least 2 h a day, during the 6-month intervention, back extensor strength increased with 27% which may indicate that the activating spinal orthosis has a positive impact on back extensor strength which has been shown in previous studies [19, 20, 25, 36, 37].

### Back pain

In our study, back pain showed no significant decrease between the three treatment groups, as opposed to some studies where the effect of treatment with an activating spinal orthosis has been studied. Our study population consisted of women suffering from osteoporosis and back pain, of whom 44% had suffered at least one vertebral fracture. There was no woman in our study suffering from an acute or sub-acute vertebral fracture. Women were also included in our study with minor symptoms of back pain and they estimated their back pain as weak (light) to very strong, with the median estimated score according to Borg CR-10 last week being 3 and VAS being 42 mm (moderate). In a prospective comparative study of rehabilitation after an acute vertebral fracture, where pain was measured with VAS and Oswestry Low Back Pain Disability Questionnaire (OLBPDQ), two types of spinal orthosis were compared, a three-point orthosis and an activating orthosis, with 140 women split into two groups wearing the orthosis for 6 months [38]. The results showed that in women wearing the activating orthosis, back pain decreased significantly (*p* < 0.05), compared to women wearing the three-point orthosis. In another prospective randomized trial, an activating spinal orthosis was compared with a soft lumbar orthosis on patients with acute or sub-acute vertebral fracture. The results showed a significant reduction in back pain but no significant difference was shown between the two orthoses [4]. In the studies by Pfeifer et al. where women suffering from a vertebral fracture for the previous 6 months were included, back pain decreased significantly by 41% after 6 months of treatment with the activating spinal orthosis [19, 20]. Dionyssiotis et al. showed that by wearing the activating orthosis for 6 months, back pain decreased with 37%. Women included in this study had had at least one vertebral fracture [25]. To conclude, in these previous studies, the populations consisted of women who were suffering from a vertebral fracture in an acute or sub-acute phase.

### Back extensor strength

In previous studies where the effect of treatment with an activating orthosis was investigated, the results showed a significant increase in back extensor strength [19, 20, 25, 36, 37]. The activating spinal orthosis has been used in rehabilitation of acute and sub-acute vertebral fractures as well as in longer term rehabilitation. In two randomized trials, back extensor strength increased by 73% and 72% respectively after 6 months’ treatment with the activating spinal orthosis [19, 20]. This increase is significantly greater than in our study where the back extensor strength increased by 27%. In the two studies, the inclusion criterion was at least one vertebral fracture occurred for the previous 6 months, and people with severe degenerative changes in the back were excluded. Inclusion and exclusion criteria may be an explanation why back extensor strength did not increase to the same extent in our study, where women were included with or without vertebral fractures and the vertebral fractures could be of older date. Women with degenerative changes in the spine and women with big differences in physical capacity and strength were also included; we excluded only persons with spinal stenosis and severe scoliosis.

Another brand of spinal orthosis (Thämert Osteo-Med) was used in a RCT in longer term rehabilitation of 72 women (mean age 74 years) with or without vertebral fractures and it was shown that after 6 months treatment, back extensor strength increased significantly in women wearing the activating orthosis compared to the control group [36].

In a prospective study where the effect of long-term use of an activating spinal orthosis was investigated in women with diagnosed osteoporosis and at least one vertebral fracture, back extensor strength increased significantly by 25% compared to the control group, after 6 months of treatment [25]. In our study, 56% of the women had no vertebral fracture but back extensor strength increased by 27% as well after 6 months of treatment, although this increase was not statistically significant compared to the controls. In order to achieve a good exercise effect with increased back extensor strength, the time wearing the activating orthosis may be important. In our study, we chose 2 h/day because in comparable studies where the result showed an increase in back extensor strength, the participants wore the activating spinal orthosis for at least 2 h/day. Time could be divided in shorter periods and they could also wear the orthosis more than 2 h/day. In the study by Meccariello, the participants wore the activating spinal orthosis for 2.5 months when sitting or standing, no specification of time was given. In two studies by Pfeifer and in one study by Dionyssiotis, the participants wore the spinal orthosis for at least 2 h/day and in another study by Li, the participants wore the spinal orthosis 3 h/day [4, 19, 20, 25, 38].

### Spinal curvature

Influence of an activating spinal orthosis on spinal curvature has been previously investigated in some studies. Pfeifer et al. showed a significant decrease in the angle of kyphosis after 6 months of treatment with the activating spinal orthosis [19, 20]. In our study, there was no significant change in spinal curvature between the three treatment groups after 6 months of treatment and there was no significant change of the kyphotic index within the respective group. Unlike the cited studies, women who participated in our study had to a greater extent chronic back problem and had developed a kyphosis a long time ago. One explanation that we did not receive a significant measurable decrease of the kyphosis may be that chronic and prolonged conditions are more difficult to influence, and there a long-term strategy and treatment plan may be needed. In a meta-analysis, the effect of treatment of orthoses has been investigated on back pain and the kyphosis angle in rehabilitation after an acute or sub-acute vertebral fracture. The results showed that treatment with the activating spinal orthosis after a sub-acute vertebral fracture significantly improved back pain and the kyphosis angle but the overall quality of the results was rated as low [39]. This may indicate that the kyphosis angle may be affected in a sub-acute stage, but it will be more difficult at a later stage. In the later stage, training of the back extensor muscle may be very important to prevent a progression of the kyphosis [11, 18, 40]. Wearing an activating spinal orthosis to strengthen the back extensor muscles and prevent progression of a kyphosis may become an alternative training method. This can be explained through biofeedback that stimulates extension of the spinal back muscles by continuously wearing the spinal orthosis, which is also given as an explanation in two RCT studies [19, 20].

### Strengths and limitations

One strength of the current study was that we investigated the use of an activating spinal orthosis in a broader perspective and whether it may be an alternative treatment method for patients with back problem of varying degrees and/or of chronic character. Another strength was that all women were living in the municipality of Stockholm, and they lived in their own accommodation and could be possible patients in Primary Health Care.

One limitation could be that it is not possible blinding the intervention for the participants and the instructors.

Future research may focus on identifying patients who would best benefit from wearing a spinal orthosis which activates the back extensor muscles. Qualitative studies can provide valuable knowledge and views about experiences of wearing an activating spinal orthosis.

## Conclusions

Wearing an activating spinal orthosis at least 2 h a day for 6 months showed no significant difference in back pain, back extensor strength, and kyphotic index compared to a control group and an equipment training group. In each-group analyses, the spinal orthosis group showed an increase in back extensor strength by 27%. Training the back extensor muscles in an activating spinal orthosis indicates that the spinal orthosis may become an alternative training method of equipment training in older women suffering from osteoporosis with or without vertebral fractures visiting Primary Health Care.

## Electronic supplementary material

ESM 1 (DOCX 16 kb)
