# Supplementary material for: Effect of treatment on back pain and back extensor strength with a spinal orthosis in older women with osteoporosis: a randomized controlled trial
Source: Arch Osteoporos. 2019 Jan 9;14(1):5. doi: 10.1007/s11657-018-0555-0 (PMC6325994; doi:10.1007/s11657-018-0555-0)
Supplement: Supplementary file 1 — (DOCX 16 kb) [file 11657_2018_555_MOESM1_ESM.docx]

**Supplement**

**Adverse outcomes during the intervention period**.

During the intervention period all participants was supposed to make notes about, for instance, adverse outcomes in a logbook. Review of the logbook showed that:

In the **training group, f**our participants suffered from some sort of infections, for instance, a cold, influenza, fever. One participant had problem with the stomach. One participant had a minor eye surgery.

Four participants got muscle or joint complaints. One participant fainted once.

In the **Spinal orthosis group,** four participants suffered from some sort of infections for instance a cold, influenza and fever. Headache, migraine and vertigo affected two participants and complaints from the stomach two participants. One participant got urinary tract infection. One participant got a metacarpal fracture and one participant got a costal fracture when cleaning. One participant got radius- and costal fractures and also a neck injury in a car accident.

In the **Control group**, eight participants suffered from some sort of infections for instance a cold, influenza, fever and also borreliosis. Three participants had problems from the stomach and three participants suffered from headache, migraine and vertigo. One participant got detachment of the retina. One participant got a vertebral fracture when cleaning the house. Urinary tract infection affected three participants and three participants got muscle or joint complaints. Two participants went through a minor surgery. One participant got allergy.

|  | **Training** | **Spinal orthosis** | **Control** |
| --- | --- | --- | --- |
| Infections | 4 | 4 | 8 |
| Gastrointestinal complaints | 1 | 2 | 3 |
| Headache, migraine |  | 1 | 2 |
| Vertigo |  | 1 | 1 |
| Pain in muscles or joints | 4 | 1 | 3 |
| Detachment of the retina |  |  | 1 |
| Vertebral fracture | 1 |  | 1 |
| Urinary tract infection |  | 1 | 3 |
| Minor eye surgery | 1 |  | 2 |
| Allergy |  |  | 1 |
| Fracture - other than vertebral |  | 3 |  |
| Syncope | 1 |  |  |
